# Consciousness and Personhood in Medical Care

**DOI:** 10.3389/fnhum.2018.00306

**Published:** 2018-08-02

**Authors:** Stefanie Blain-Moraes, Eric Racine, George A. Mashour

**Affiliations:** ^1^School of Physical and Occupational Therapy, McGill University, Montreal, QC, Canada; ^2^Institut de Recherches Cliniques de Montréal, Montreal, QC, Canada; ^3^Department of Anesthesiology, Center for Consciousness Science, University of Michigan Medical School, Ann Arbor, MI, United States

**Keywords:** consciousness, personhood, unresponsive wakefulness syndrome, minimally conscious state, general anesthesia, neocortical death

## Abstract

Current paradigms in Western medicine often fail to differentiate clearly between consciousness, responsiveness and personhood. The growing number of individuals who exist with sustainable cardiopulmonary systems but who are behaviorally unresponsive has prompted a cultural reconsideration of the relationship between the presence of consciousness and what it means to be a person. This article presents relevant clinical situations that exemplify the different modes in which personhood and consciousness can be associated and dissociated: disorders of consciousness, emergence from anesthesia, and neocortical death. We draw from these examples to call for a reflection on and possible revision of the dominant approach towards unresponsive persons to one in which care providers may work from the default assumption of the existence of an individual’s personhood as part of their therapeutic intervention. Behavior consistent with this assumption aligns with the principle of respect for persons in the face of the uncertainty created by the high rate of misdiagnosis of unconsciousness in unresponsive patients and is most consistent with a therapeutic approach to care considering evidence suggesting that attributing personhood may in fact evoke consciousness in these patients.

## Introduction

The concepts of person and personhood play pivotal roles in Western medical care and clinical ethics (Gordon, 1934; Ramsey, 1970). The claim to personhood has long been considered to be threshold-setting such that, when someone is considered a person, a number of entitlements and obligations follow that are not attributable to non-persons. Advances in life-saving technology are increasing the number of individuals who exist with little to no responsiveness, and their existence troubles our notions of consciousness, responsiveness and personhood, as well as their ethical implications. In the past, unresponsive patients were considered to lack core features of personhood. This meant that, for some care providers, these patients were in a moribund state such that justified an attitude described as “therapeutic nihilism” (Fins, 2015). However, the equation was not direct or necessarily explicit since most scholars and professional associations argued that unresponsive persons nevertheless needed to be treated with respect (respect for persons). Nevertheless, research has shown that attitudes toward those patients implied dismissive opinions perhaps equally consistent with a form of non-respect of persons (Kuehlmeyer et al., 2014; Fins, 2015; Byram et al., 2016). Importantly, these attitudes are not found solely or necessarily predominantly in clinicians and may be widespread in the public of Western societies (Gray et al., 2011).

In this article, we draw from examples of relevant clinical situations (e.g., unresponsive wakefulness syndrome (UWS), emergence from anesthesia, and neocortical death) to call for reflection on and a possible revision of approaches towards unresponsive persons to one in which care providers may work from the default assumption of the existence of an individual’s personhood as part of their therapeutic intervention. Behavior consistent with this assumption aligns with the principle of respect for persons in the face of the uncertainty caused by the high rate of misdiagnosis in unresponsive patients, and; is most consistent with a therapeutic approach to care considering preliminary studies suggesting that attributing personhood may in fact evoke consciousness from individuals in this population.

## The Evolving Relationship Between Consciousness, Personhood and Responsiveness

Conceptually, consciousness and personhood are distinct entities. Consciousness has been described as “what abandons us every night when we fall into dreamless sleep” (Searle, 1998) and entails subjective experience. This subjective experience can be connected to stimuli from an individual’s immediate environment, or can be disconnected, as in the cases of dreaming (Stickgold et al., 2001) and some forms of anesthesia (Domino, 2010). Whether or not consciousness is connected or disconnected, its presence or absence is not predicated upon the presence of, attitudes of, or types of interactions with other individuals.

Personhood is “a standing or status that is bestowed upon one human being, by others, in the context of relationship and social being” (Kitwood, 1997). In contrast to consciousness, personhood arises from relationships between two human beings and thus is entirely dependent on their interpersonal interactions as well as the attitudes that both individuals bring towards each other in their relationship. The accordance of personhood to another individual leads to entitlements and obligations within the human community/community of human beings. Although consciousness and personhood are thus clearly independent constructs, the two concepts are so interwoven in everyday interactions (where both are present) and in perceptions of death (where both are absent) that they appear to be inextricably linked, such that to display signs of consciousness is to have personhood.

Advances in respiratory technology in the 1940s and 50s enabled the unprecedented survival of individuals whose traumas and pathologies would have otherwise been fatal. As these life-saving technologies became integrated into routine medical practice, “new kinds of persons and modes of life” (Kaufman, 2005, 84) were inadvertently created in the form of individuals whose physiological function was maintained by machines yet who exhibited no sign of responsiveness. Puzzled by the state of these patients, Mollaret and Goulon (1959) described this condition as “beyond coma” (“coma dépassé”). In response to escalating uneasiness about the ethical implications of this complex situation, notably the lack of clarity of these patients, an *ad hoc* committee was convened under the leadership of Henry Beecher at the Harvard Medical School, which published a landmark article in 1968 on the neurological criteria for death determination. The committee proposed that irreversible coma be considered as an acceptable criterion for death, the corollary of which was that the “person” could be dead at the same time that internal organs could be kept viable by machines or other forms of support (*Ad Hoc* Committee of the Harvard Medical School to Examine the Definition of Death, 1968). This new criterion for determining death addressed the status of personhood for a category of individuals with severe brain injury; however, standards for attributing consciousness and personhood to other unresponsive individuals remained ambiguous. For example, providing the first account of the vegetative state (VS) Jennett and Plum (1972) described these patients as “mindless.” That account of the VS/UWS was incorporated in the practice guidelines of major neurological societies (American Academy of Neurology, 1989; The Quality Standards Subcommittee of the American Academy of Neurology, 1995; Royal College of Physicians, 2003), but remained contested by critics and some family members. Tensions about the ambiguity of the lack of awareness about these patients centered on the discrepancy between the diagnosis of unconsciousness and the presence of so-called reflexive behavior, such as spontaneous eye opening. These tensions only increased as an fMRI case report claimed that unresponsive individuals could possess “covert consciousness” (Owen et al., 2006; Mashour and Avidan, 2013). This finding, originally intensely debated, has become increasingly accepted with larger studies and a number of confirmatory studies (Monti et al., 2010). A case-control study demonstrated that specific damage to motor thalamocortical fibers could explain the dissociation between preserved covert behavior and awareness and the absence of overt motor behavior in such patients, highlighting a possible mechanism for the dissociation between consciousness and responsiveness (Fernández-Espejo et al., 2015). At this time, since a direct measure of subjective experience currently does not exist and advanced neuroimaging remains mostly a research tool, inferences of consciousness are made based on responsiveness as observed by clinicians. However, individuals who are comatose, in UWS, minimally conscious state, and the locked-in syndrome may exhibit similarly low levels of responsiveness while having dramatically different levels of consciousness.

While consciousness remains a subjective phenomenon whose recognition by others can depend a person’s behavioral repertoire, personhood is a relational phenomenon, in that it is *attributed* to one individual by another, and is dependent upon if one possesses a set of characteristics defined by the attributor (Pilapil, 2012; Demertzi et al., 2013). As such, one’s personhood status can be modified not only by a change in these characteristics, but by a change in an attributor’s set or definition of these characteristics. While personhood is typically attributed on the basis of an individual’s state of consciousness (Cranford and Smith, 1987), the next three sections present examples where consciousness and personhood are dissociated, in order to enable an exploration of the relationship between these two concepts.

### Personhood Without Consciousness: Unresponsive Wakefulness Syndrome

Individuals in UWS, formerly known as VS, are deemed to be unresponsive and unaware. In other words, despite the retention of some reflexive behaviors such as eye opening and yawning, these individuals lack consciousness. This lack of conscious awareness has led to some attitudes and behaviors implying that these individuals are not persons anymore. Indeed, the activities surrounding these individuals are a cultural experiment in ways of knowing and defining what *a person* is (Kaufman, 2005). Studies of North American hospital units where health care workers and relatives interface with patients in UWS show how this growing population of patients without consciousness troubles our understanding of life and death, and challenges Western notions of personhood (Kaufman, 2005). In clinical units with patients whose consciousness seems remote or absent, personhood is defined primarily through caregivers’ discussion of the body and its regulation, and through social elements such as concern, empathy, responsibility and interpretation. Although patient responsiveness and consciousness are helpful, they are not essential for the determination of personhood (Kaufman, 2005, 290). To family members, patients in UWS are seen in the context of their life history, pre-illness personality and relationships. Although they may not move or respond, these patients are considered by their families as *whole*, though physically vulnerable, persons (Kaufman, 2005, 299). This attitude is mirrored in current treatment of individuals with advanced dementia, who may be unresponsive and unable to provide evidence of consciousness, yet are accorded personhood by their care providers (Kitwood, 1997; Volicer et al., 1997). The dissociation of personhood and consciousness is not limited to family and caregivers of unresponsive individuals. A survey of neurologists and medical directors of nursing homes regarding their attitudes towards patients in UWS reported that even those with the most experience in observing and treating this population give contradictory assessments of consciousness and personhood (Payne et al., 1996). In a Canadian-German survey, it was found that most specialty physicians attributed pain perception to patients in UWS and significant proportion in in both countries attributed touch, smell, hunger and thirst to those patients (Kuehlmeyer et al., 2014). These results reveal an overall confusion about the nature of consciousness and ambivalence about what constitutes the *person*. The definition *a person* is also influenced by the cultural background of the attributor or interaction partner. Individuals from collectivists societies, where relationships among people are prioritized, often have different thresholds for the determination of personhood than those from individualistic societies, which promote the development of individual values. For example, in Japan (highly collectivistic), an individual is conceptualized as residing within a network of obligations. In this culture, personhood is not constructed from an individual’s physical or mental capacity, but rather in the space of ongoing human relationships. As such, “person” is a dialogical creation, and even a brain dead body is still a person because its social identity remains intact (Lock, 2000, 256).

### Consciousness Without Personhood: Emergence From Anesthesia

In the operating room, patients are transformed from subjects of concern to objects of attention after being anesthetized (Katz, 1981). Indeed, the very purpose of general anesthesia is the reversible extinction of subjectivity such that the body can be manipulated purely as an object; this differs notably from the state of sleep, in which personhood or even consciousness (in the case of dreams) is considered preserved. While anesthetized patients are unconscious, they are typically discussed using pronouns (rather than proper names) and metonymy (the substitution of one word for a related concept)—terms that are medically efficient, but minimize patient personhood (Kopp and Shafer, 2000). Upon emergence, consciousness is regained, but the return of personhood can lag in comparison. Although it is recommended that patients emerging from anesthesia be allowed to do so in a calm quiet environment with reassurance (Smith and Mishra, 2010), as during induction (Smith et al., 2005), the period of emergence is often noisy from extraneous conversation. We speculate that this common phenomenon is due to a persistent perception of the patient as an insensate (or at least amnesic) object, despite the return of subjectivity. In other words, consciousness has recovered but personhood is not yet fully recognized.

### Equivalence of Personhood and Consciousness in Disease States: Neocortical Death

Neuroimaging technologies have enabled scientists to identify activities and interactions in the brain related to consciousness, even without behavioral responsiveness (Rees et al., 2002; Monti et al., 2010; Dehaene and Changeux, 2011; Casali et al., 2013; Voss et al., 2014). This practice has given rise to a dominant biomedical ontology of consciousness that reifies consciousness as a “thing” to be detected and seen in the brain (Nettleton et al., 2014). Using these scientific findings as support, some neurologists and philosophers have argued that permanent cessation of “those higher functions of the nervous system that demarcate man from the lower primates” (Brierley et al., 1971)—i.e., neocortical function—be used as the criteria for defining death. The conceptual basis for their argument rests on the premise that consciousness, cognition and social interaction are the essential characteristics of human life (Veatch, 1975; Gervais, 1988). For those who hold this perspective, the concepts of consciousness and personhood are synonymous; consequently, personhood is considered extinguished in those without neural evidence of consciousness, such as individuals in UWS and anencephalic infants (Laureys, 2005).

## Implications for Clinical Practice

As unresponsive individuals become more prevalent in acute and long-term settings, the associations and dissociations between responsiveness, consciousness and personhood described above have garnered increasing social and media attention, such as in the tragic case of Terri Schiavo (Annas, 2005; Quill, 2005; Racine et al., 2008). Situations in which the medical assessment of an unresponsive patient’s level of consciousness have not aligned with the family’s understanding of that patient’s personhood have led to public disputes about treatment decisions as well as lengthy and costly litigation (Pence, 2004). Conscious individuals in healthcare settings who are not attributed personhood are at high risk of post-traumatic stress disorder, and report feelings of frustration, panic, anxiety and dehumanization (Happ, 2000). Caregivers of unresponsive patients also report that discrepancies between medical assessments of the patient’s capacity for consciousness and their own perceptions of the patient’s personhood are a source of profound distress (Happ, 2000). As the dissociation between personhood and consciousness becomes increasingly problematic in healthcare settings, healthcare professionals must assume a leadership role in guiding society’s understanding and attitudes of the relationship between these two concepts. The approach that physicians and nurses take toward personhood in an unresponsive patient can/could influence the prognosis and experiences of these individuals, and may affect end-of-life decisions (Demertzi et al., 2011). It is critical that these approaches be adopted intentionally and deliberately. Survey research suggests that in current practice, most healthcare providers believe that the withdrawal of care is the most ethical decision to make in the treatment of these individuals (Kuehlmeyer et al., 2014). We suggest that the default position of care providers should be the attribution of personhood to unresponsive individuals, regardless of evidence of consciousness: because (1) there are high rates of misdiagnosing unconsciousness; and (2) attributing personhood to an individual may potentially facilitate the recovery of consciousness. Importantly, attribution of personhood is not the only consideration for ethical decision-making since the benefits of maintaining care and the patient’s preferences are crucial considerations.

Our current ability to detect consciousness in unresponsive patients is still in its infancy. Without formal behavioral assessment, up to 41% of neurologically-impaired patients assumed to be unconscious are erroneously diagnosed as being in a “VS” when in fact they are in a minimally conscious state (Schnakers et al., 2009). In other words, the presence of consciousness goes clinically undetected in almost half of patients who cannot reliably respond to the external environment. A study using functional neuroimaging demonstrated that even among those patients who are formally diagnosed with UWS, 17% are able to respond to commands by activating specific brain regions, and are therefore argued to be conscious (Monti et al., 2010). A separate large-scale study using transcranial magnetic stimulation to assess the complexity of EEG responsiveness showed that 9 of 43 patients in UWS had brain responses similar to those who could subjectively report their conscious awareness (Casarotto et al., 2016). These studies likely underrepresent the number of individuals who exist with undetected consciousness (Bardin et al., 2011). Furthermore, during what is assumed to be adequate general anesthesia, 1–2 patients per 1000 have been reported to experience and remember the events of surgery (Mashour et al., 2012); this incidence rises to approximately 1% in high-risk patients (Avidan et al., 2011). Conservatively speaking, around 5% of surgical patients have consciousness of intraoperative events without memory (Sander et al., 2017), which increases to 40% in some situations (Sanders et al., 2012).

The attribution of personhood to nonresponsive individuals not only satisfies the moral imperative of according those with “covert consciousness” the rights of human beings, it also may facilitate positive outcomes. Although sensory stimulation paradigms have a long history of being used in neurorehabilitation (Schnakers et al., 2016), recent evidence has demonstrated that stimulation that acknowledges an individual’s history and interpersonal connections may be more effective at evoking consciousness. A crossover treatment design study in a patient with chronic traumatic brain injury demonstrated that administration of biographically relevant stories narrative by familiar voices caused increased behavioral responses compared to baseline, and that the degree of responsiveness decreased during the subsequent 6-week sham period (Sullivan et al., 2018). The same group used a double-blind randomized placebo-controlled trial with individuals in minimally conscious state or with UWS (*n* = 15) to test the effect of familiar people telling stories about memorable events in comparison to placebo silence (Pape et al., 2015). Patients in the experimental group had significantly quicker recovery and higher function than the control group. This may relate to activation of higher-order cortical regions, since fMRI analysis demonstrated significantly increased prefrontal cortex activity during exposure to the familiar voice in the experimental group. In a related study, nine coma patients with traumatic brain injury were exposed to sounds that were either personally relevant or personally irrelevant. Although both types of stimulation increased arousal in comparison to baseline, the changes were significantly greater if the sounds were personally relevant (Park and Davis, 2016). Although research in larger cohorts is necessary to validate these findings, these results suggest that the acknowledgment of an individual’s interpersonal connections and emotionally-salient memories—in other words, acknowledgment of an individual’s personhood—may significantly influence an individual’s level of recovery of consciousness.

Aside from this potential benefit of attributing personhood in unresponsive states, it is important to note that this recommendation fits within standard medical ethical principles (Beauchamp and Childress, 2012). Our recommendation of recognizing personhood does not undermine the ability for an individual to make their own treatment and care decisions. As such, it does not entail an alignment with a stance in favor of maintaining life-sustaining interventions despite the patient’s or the family’s wishes. Our recommendation is instead related to the caring and just attitude that healthcare providers should adopt when there is uncertainty. Assuming that the patient and/or the family want to pursue care, what practices are the most beneficent, least harmful, least prejudging of the ability to recover and least prejudging of the value of a life with disability? The beneficent obligation that healthcare providers have in these scenarios calls for the creation of a care ethos that reflects the principle of respect of persons for these patients.

It is also important to make clear that our call to assume personhood in unresponsive individuals in healthcare settings does not extend to those who are dead. Once death has been determined, by neurological or cardiovascular criteria, it is crucial that healthcare providers communicate that the ultimate loss of personhood has occurred. The consequences of not clearly communicating this message are costly from both emotional and financial perspectives (Dasta et al., 2005; Salim et al., 2005; Murugan et al., 2009; McAdam et al., 2010; Arbour, 2012; Curtis et al., 2012; McKeown et al., 2012).

## Conclusion

Health care professionals play a pivotal role in shaping society’s understanding of and response to the complex relationship between consciousness and what it means to be a person. We have illustrated through various examples that consciousness and personhood are in fact doubly dissociable. However, given that this dissociation exists outside of everyday experience, it behooves those who regularly interact with unresponsive patients to set the standard for negotiating the relationship between these two concepts. We advocate that healthcare providers work from the default assumption of the existence of an individual’s personhood as part of their therapeutic practice. In light of the uncertainty around the presence vs. absence of consciousness of unresponsive individuals, the assumption of personhood is consistent with the principle of respect for persons. There is also emerging evidence that behavior consistent with this assumption may in fact facilitate the recovery of consciousness in those without it, supporting the attribution of personhood as a preferred therapeutic approach to care. However, the attribution of personhood must be consistently re-evaluated in light of emerging medical and scientific data, such that when new evidence is presented (e.g., evidence of brain death), an individual’s status of personhood can be adjusted accordingly. Also, prior wishes of patients not wanting to be sustained in UWS should be respected.

## Author Contributions

SB-M, ER and GM all contributed to the formation of the ideas presented in this article. All authors contributed to the writing and revision of the manuscript.

## Conflict of Interest Statement

The authors declare that the research was conducted in the absence of any commercial or financial relationships that could be construed as a potential conflict of interest.
